# Relationships Between Markers of Inflammation and Muscle Mass, Strength and Function: Findings from the Hertfordshire Cohort Study

**DOI:** 10.1007/s00223-017-0354-4

**Published:** 2017-11-03

**Authors:** L. D. Westbury, N. R. Fuggle, H. E. Syddall, N. A. Duggal, S. C. Shaw, K. Maslin, E. M. Dennison, J. M. Lord, C. Cooper

**Affiliations:** 1 MRC Lifecourse Epidemiology Unit, Southampton General Hospital, University of Southampton, Southampton, SO16 6YD UK; 2 MRC-ARUK Centre for Musculoskeletal Ageing Research, Institute of Inflammation and Ageing, University of Birmingham, Birmingham, UK; 3 Victoria University of Wellington, Wellington, New Zealand; 4 NIHR Southampton Biomedical Research Centre, University of Southampton and University Hospital Southampton NHS Foundation Trust, Southampton, UK; 5 NIHR Musculoskeletal Biomedical Research Unit, University of Oxford, Oxford, UK

**Keywords:** Inflammation, Muscle, Sarcopenia, Strength, Adipokine, Interleukin

## Abstract

We investigated the longitudinal relationships between inflammation markers and the following outcomes in a UK cohort study: appendicular lean mass (ALM); walking speed; level and change in grip strength; and sarcopenia defined by the European Working Group on Sarcopenia in Older People. Analyses were based on 336 community-dwelling older men and women (aged 59–70 years) who participated in the Hertfordshire Cohort Study (HCS). Inflammation markers were ascertained at baseline using enzyme-linked immunosorbent assay techniques and Bio-Plex Pro Assays. Grip strength was measured at baseline and follow-up [median follow-up time: 10.8 years (inter-quartile range 10.2–11.6)] and change in grip strength was ascertained using a residual change approach. At follow-up, ALM was ascertained using dual-energy X-ray absorptiometry, customary walking speed was measured and sarcopenia status was ascertained. Gender-adjusted linear and Poisson regression was used to examine the associations between inflammation markers and outcomes with and without adjustment for anthropometric and lifestyle factors. Higher C-reactive protein was associated (*p* < 0.04) with lower grip strength and accelerated decline in grip strength from baseline to follow-up. Higher cortisol was associated with lower ALM (*p* < 0.05). Higher interleukin-8 (IL-8) was associated with lower ALM (*p* < 0.05) and increased risk of sarcopenia [fully-adjusted relative risk per SD increase in IL-8: 1.37 (95% CI 1.10, 1.71), *p* = 0.005]. All associations were robust in fully-adjusted analyses. Inflammation markers were associated with measures of muscle mass, strength and function in HCS. Further work is required to replicate these associations and to delineate the underlying mechanisms.

## Introduction

Sarcopenia is an age-related syndrome characterised by loss of skeletal muscle mass and strength. It is a major contributor to the risk of physical frailty, functional impairment, poor health-related quality of life, and premature death in older people [1]. The prevalence varies [2] and it is associated with an estimated annual cost of £2.5 billion to the UK health economy [3].

Previous evidence supports the assertion that immunosenescence leads to a chronic, low-grade inflammatory state which contributes to cellular ageing, and an increased susceptibility to infection, autoimmunity and neoplasia [4]. A relationship may exist between inflammation and muscular ageing through a process of catabolism and skeletal muscle atrophy [5].

Interleukin-6 (IL-6), interleukin-1 receptor (IL-1R), tumour necrosis factor (TNF) and C-reactive protein (CRP) are primary elements in the inflammatory cascade which have been associated with disability [6], poor physical performance [7], reduced muscular strength [8] and muscle mass [9]. However, a recent meta-analysis of the relationship between three markers of inflammation and sarcopenia only showed a relationship with CRP but none with TNF or IL-6 [10]. This inconsistency highlights the need for further research which investigates the relationships between a wider range of inflammatory markers and sarcopenia.

The Hertfordshire Cohort Study (HCS) [11] provides an excellent opportunity to investigate these associations among a population-based cohort of community-dwelling older men and women from the United Kingdom.

The objective of this study was to investigate the longitudinal associations between a wide range of markers of inflammation and the following outcomes among HCS participants: appendicular lean mass (ALM), walking speed, level and change in grip strength, and sarcopenia defined according to the European Working Group on Sarcopenia in Older People (EWGSOP) consensus algorithm [1].

## Methods

### The Hertfordshire Cohort Study

The HCS comprises 1579 men and 1418 women born in Hertfordshire between 1931 and 1939 and who still lived there in 1998–2004 when they completed a baseline home interview and attended a research clinic for detailed characterisation of their socio-demographic, lifestyle and clinical characteristics; the study has been described in detail previously [11].

Smoking status, weekly consumption of alcohol and level of physical activity (Dallosso questionnaire [12]) were ascertained by a nurse-administered questionnaire at the home interview. Participants completed a food-frequency questionnaire from which a ‘prudent diet’ score was derived using principal components analysis; higher scores reflect healthier diets [13]. Details of all prescription and over-the-counter medications currently taken were coded according to the British National Formulary; the number of systems medicated was used as a marker of comorbidity.

Investigations conducted at the baseline clinic included measurement of height (using a Harpenden pocket stadiometer, Chasmors Ltd, London, UK) and weight (on a SECA floor scale, Chasmors Ltd, London, UK). Grip strength was assessed three times for each hand using a Jamar dynamometer; the highest measurement was used for analysis. Blood samples were taken and serum was aliquoted and stored at − 80 °C.

Overall, 966 participants resident in East Hertfordshire underwent a dual-energy X-ray absorptiometry (DXA) scan at baseline. In 2004, 642 of them were recruited to a musculoskeletal follow-up study. In 2011, 591 were invited to participate in a further follow-up study; 443 agreed to participate [median follow-up time from baseline 10.8 years (inter-quartile range 10.2–11.6)] [14]. Grip strength and height were measured using the same protocol as at baseline, mean customary walking speed in metres per second was ascertained using the mean time from two 8ft gait speed tests, and ALM was derived using DXA (Lunar Prodigy Advanced Scanner, GE Medical Systems, UK).

**Fig. 1:**
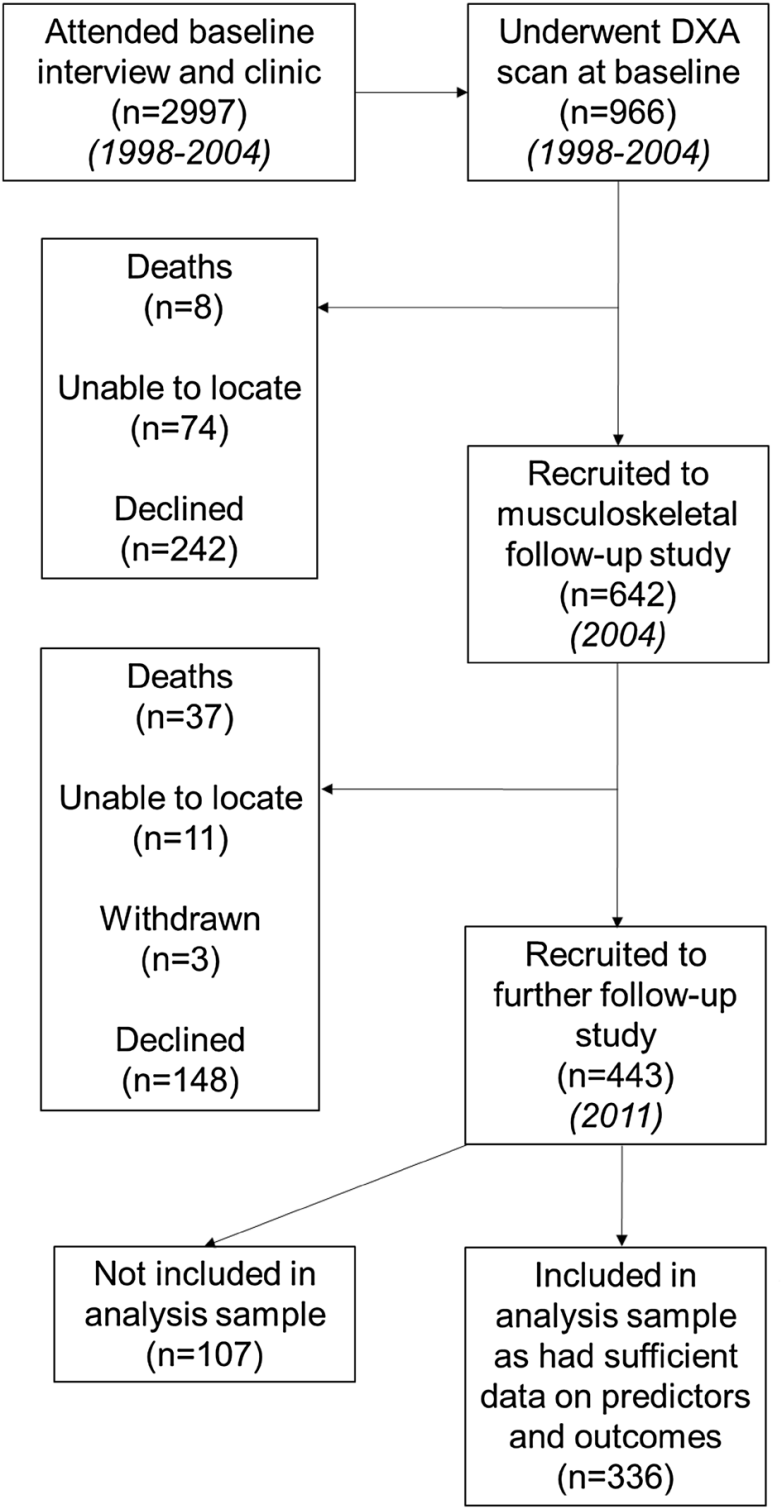
Flow diagram for the Hertfordshire Cohort Study analysis sample

The HCS analysis sample for this paper comprised 336 participants who: had attended the 2011 follow-up stage of HCS, had non-missing data for at least one of the inflammation markers considered; and had at least one non-missing value for each of the outcomes. A flow diagram for the HCS analysis sample is presented in Fig. 1.

### Ascertainment of Baseline Inflammatory Markers

Serum cortisol, dehydroepiandrosterone sulphate (DHEAS) and CRP levels were measured by enzyme-linked immunosorbent assay (ELISA) techniques using a commercial kit (IBL international, Hamburg, Germany) according to manufacturer’s instructions. Plasma adiponectin (serum diluted 1:5000) and leptin (serum diluted 1:2000) were assessed separately by solid phase sandwich ELISA (R&D Systems, Abingdon, UK). Data analysis was done using GraphPad Prism software (GraphPad Software Ltd, USA).

A multiplex based assay for the cytokines interleukin-10 (IL-10), interleukin-8 (IL-8), IL-6 and TNF (Bio-Rad Laboratories, Munich, Germany) was performed according to manufacturer’s instructions. Data acquisition and analysis was conducted using Bio-Plex Manager software version 6.0.

### Derived Measures

Height and weight were highly correlated (*r* = 0.43, *p* < 0.001 for men; *r* = 0.39, *p* < 0.001 for women); to avoid multi-collinearity problems, a sex-specific standardised residual of weight-adjusted-for-height at baseline was derived. Conditional change in grip strength was characterised by residuals obtained after estimating sex-specific linear regression models for grip strength at follow-up on grip strength at baseline with adjustment for individual follow-up duration; this measure of change is independent of baseline level. Appendicular lean mass index was calculated by dividing ALM (kg) by the square of height (m). Sarcopenia status at follow-up was derived using the EWGSOP sarcopenia algorithm [2] with the following cut-points: ALM index (ALM/height^2^) ≤ 7.23 kg/m^2^ for men (≤ 5.67 kg/m^2^ for women); grip strength < 30 kg for men (< 20 kg for women); and walking speed ≤ 0.8 m/s. Participants with slow walking speed or weak grip strength, and who also had low ALM index were classed as sarcopenic.

### Statistical Methods

Summary statistics were used to describe the data. Apart from IL-8, all inflammatory markers were highly positively skewed and were log-transformed. Linear regression was used to examine the longitudinal association between each baseline inflammatory marker and the following continuous outcomes: walking speed, grip strength and ALM at follow-up; and conditional change in grip strength from baseline to follow-up. The association between baseline inflammatory markers and sarcopenia status at follow-up was examined using Poisson regression models with robust variance estimation to yield relative risks. Gender-adjusted and fully-adjusted models, which also accounted for gender, baseline age, follow-up time, height, weight-for-height, smoking history, alcohol consumption, prudent diet score and physical activity were implemented.

Sex-specific standard deviation scores were coded for inflammatory markers and outcomes and used in models. To maintain sample size, men and women were pooled and analyses were adjusted for gender; *p* < 0.05 was regarded as statistically significant. Analyses were conducted using Stata, release 13.

## Results

### Participant Characteristics

Characteristics of the 336 HCS participants who were included in the analysis sample are presented in Table 1. Mean (SD) age at HCS baseline was 63.8 (2.5) and 65.6 (2.7) years among men and women, respectively. Median (inter-quartile range) time from HCS baseline clinic to the 2011 follow-up was 11.6 (11.1, 11.9) years among men and 10.1 (9.8, 10.4) years among women. On average, men had higher ALM and faster walking speed compared with women. On average, men had much higher grip strength than women at both baseline and follow-up but experienced greater annual loss of grip strength (mean (SD) change in grip among men and women, respectively: − 0.71 (0.48) kg/year vs − 0.58 (0.49) kg/year).

**Table 1:** Characteristics of 336 participants from the Hertfordshire Cohort Study

Mean (SD)	Men (*n* = 186)	Women (*n* = 150)	Obs
Age at HCS baseline clinic (years)	63.8 (2.5)	65.6 (2.7)	336
Follow-up time (years)^a^	11.6 (11.1, 11.9)	10.1 (9.8, 10.4)	336
Height (cm)	174.7 (6.5)	161.7 (5.5)	336
Weight (kg)	80.8 (11.0)	69.2 (12.2)	336
BMI (kg/m^2^)	26.5 (3.3)	26.4 (4.3)	336
Ever smoked^b^	113 (60.8%)	50 (33.3%)	336
Alcohol consumer (≥ 1 unit per week)^b^	166 (89.2%)	86 (57.3%)	336
Prudent diet score	− 0.6 (2.0)	1.1 (1.8)	336
Dallosso physical activity score	65.0 (13.5)	63.0 (14.1)	336
Number of systems medicated^a^	1 (0, 1)	1 (0, 2)	336
CRP (mg/L)^a^	0.7 (0.4, 1.9)	1.1 (0.5, 2.3)	322
Adiponectin (ug/ml):Leptin (ng/ml) ratio^a^	0.6 (0.2, 1.5)	0.2 (0.1, 0.4)	322
IL-10 (pg/ml)^a^	7.0 (5.9, 10.6)	7.2 (5.9, 10.6)	334
IL-6 (pg/ml)^a^	6.2 (3.4, 8.8)	6.1 (3.1, 7.6)	286
Cortisol (ug/ml)^a^	0.10 (0.09, 0.12)	0.09 (0.08, 0.10)	331
DHEAS (ug/ml)^a^	1.1 (0.8, 1.5)	0.7 (0.5, 1.0)	333
TNF (pg/ml)^a^	4.7 (2.5, 11.6)	3.5 (2.9, 9.8)	323
IL-8 (pg/ml)^a^	7.5 (5.4, 10.5)	6.9 (5.8, 9.2)	268
Baseline grip strength (kg)	44.6 (7.2)	27.5 (5.2)	335
Grip change (kg/year)	− 0.71 (0.48)	− 0.58 (0.49)	335
Characteristics at follow-up			
Grip strength (kg)	36.4 (7.4)	21.7 (6.1)	335
Appendicular lean mass (kg)	24.3 (2.8)	16.5 (2.2)	289
Appendicular lean mass index (kg/m^2^)	8.05 (0.75)	6.42 (0.72)	289
Walking speed (m/s)	0.79 (0.17)	0.74 (0.18)	314
EWGSOP sarcopenia^b^	12 (7.4%)	11 (8.7%)	289

*Grip change* annual change in grip from HCS baseline to 2011 follow-up, *EWGSOP* European Working Group on Sarcopenia in Older People, *Obs* number of non-missing observations

^a^Median (lower quartile, upper quartile)

^b^ *n* (%)

### Assessing Healthy Participant Effects in Analysis Sample

Compared to the 2661 participants who attended the HCS baseline clinic but were not included in the analysis sample, both men and women in the analysis sample had higher self-reported activity (Dallosso questionnaire); diet quality was higher among women only (*p* < 0.003 for both). However, smoking prevalence, alcohol consumption and the proportion who were of manual social class (classes IIIM, IV and V from the 1990 Office of Population Censuses and Surveys (OPCS) Standard Occupational Classification (SOC90) unit group for occupation [15]) did not differ significantly (*p* > 0.05) between the two groups; this was the case among men and women.

Compared to the 107 participants who were invited to participate in the follow-up study in 2011 but were not included in the analysis sample, men in the analysis sample had higher self-reported activity (*p* = 0.01); there were no differences in other health behaviours or occupation class among men or among women.

### Associations Between Baseline Inflammation Markers and Muscle Mass, Strength and Function at Follow-Up

**Fig. 2:**
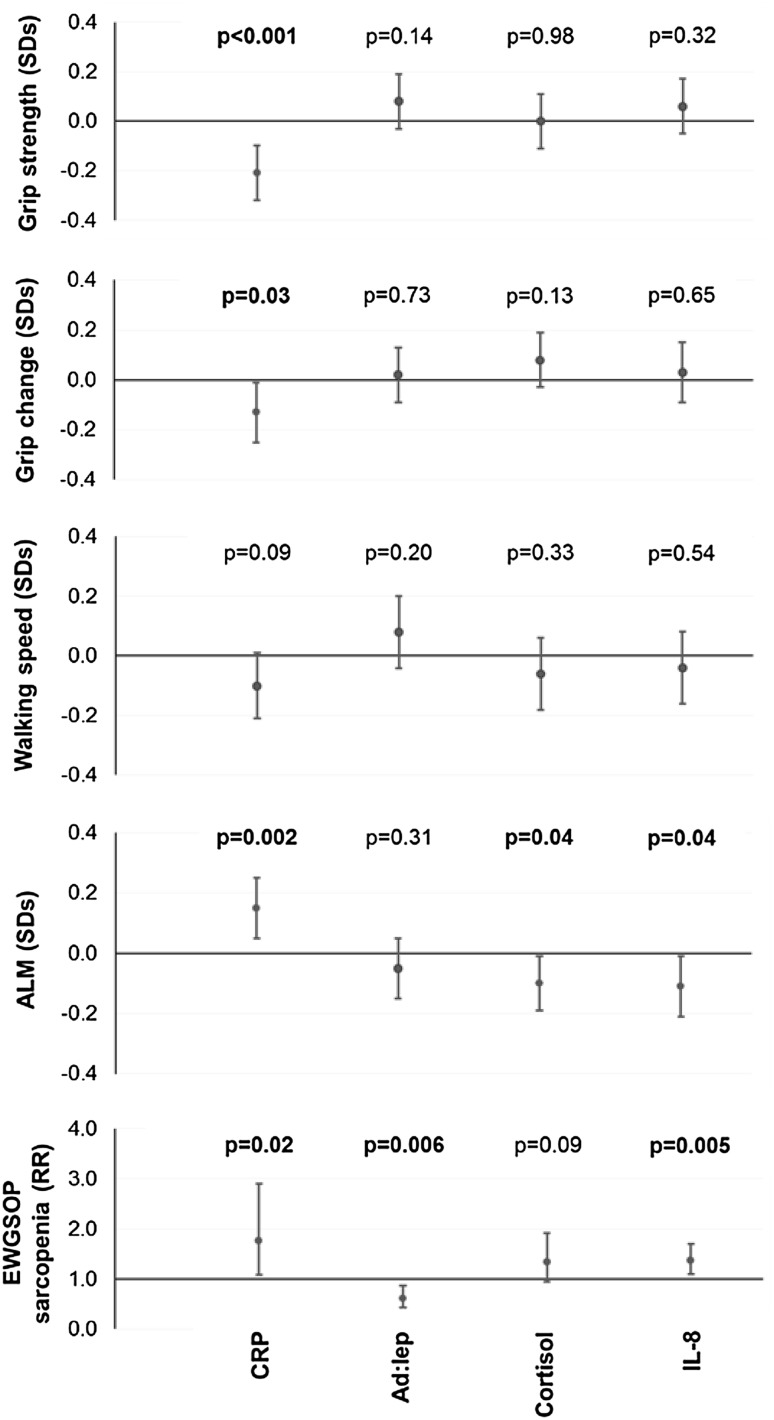
SD difference (95% CI) in outcomes per SD increase in inflammatory predictors. *Ad:lep* adiponectin:leptin ratio. Regression models adjusted for the following characteristics at baseline: gender, age, follow-up time, height, weight-for-height residual, smoking history (ever vs never), alcohol consumption, diet quality and physical activity. Poisson regression models with robust variance estimation were used for EWGSOP sarcopenia status to yield relative risks (RR). Models for ALM were not adjusted for weight-for-height residual and models for change in grip were not adjusted for follow-up time (follow-up time was already used to derive the grip change measure). Adjusted *p* values are presented. Change in grip strength from HCS baseline to follow-up was obtained using a residual change method to ensure grip change measure was independent of baseline grip level. A positive estimate illustrates that a higher level of the inflammatory marker was associated with reduced loss of grip strength and a negative estimate reflects accelerated loss. Apart from IL-8, all inflammatory markers were log-transformed prior to standardising

Associations between baseline inflammation markers and the following outcomes are presented in Table 2 and Fig. 2: grip strength, walking speed, ALM and sarcopenia status at follow-up; and conditional change in grip from baseline to follow-up. In gender- and fully-adjusted analyses, higher CRP was associated with lower grip strength, accelerated loss of grip strength, and higher ALM. For example, an SD increase in CRP was associated with an average reduction in grip strength level of 0.21 (95% CI 0.10, 0.32) SDs. Higher CRP and lower adiponectin:leptin ratios were each associated with slower walking speed in gender-adjusted analyses only. In gender- and fully-adjusted analyses, higher cortisol and IL-8 were additional predictors of lower ALM. Higher DHEAS was only associated with lower ALM in gender-adjusted analysis.

**Table 2:** SD difference (95% CI) in outcome at follow-up per SD increase in inflammatory predictor

Outcome	Inflammatory predictor	Gender-adjusted		Fully-adjusted^a^	
		Estimate (95% CI)	*p* value	Estimate (95% CI)	*p* value
Grip strength	CRP	− **0.21 (**− **0.32,** − **0.10)**	**<** **0.001**	− **0.21 (**− **0.32,** − **0.10)**	**<** **0.001**
	Ad:lep ratio	0.05 (− 0.06, 0.16)	0.364	0.08 (− 0.03, 0.19)	0.135
	IL-10	0.05 (− 0.06, 0.16)	0.354	0.03 (− 0.07, 0.13)	0.529
	IL-6	− 0.02 (− 0.14, 0.10)	0.771	− 0.02 (− 0.13, 0.09)	0.725
	Cortisol	− 0.00 (− 0.11, 0.10)	0.930	0.00 (− 0.10, 0.11)	0.976
	DHEAS	− 0.04 (− 0.15, 0.06)	0.425	− 0.02 (− 0.12, 0.09)	0.738
	TNF	0.03 (− 0.08, 0.14)	0.543	0.03 (− 0.07, 0.13)	0.587
	IL-8	0.00 (− 0.12, 0.12)	0.945	0.06 (− 0.06, 0.17)	0.322
Grip strength change	CRP	− **0.15 (**− **0.26,** − **0.04)**	**0.006**	− **0.13 (**− **0.24,** − **0.01)**	**0.030**
	Ad:lep ratio	0.02 (− 0.08, 0.13)	0.686	0.02 (− 0.09, 0.13)	0.734
	IL-10	0.07 (− 0.04, 0.17)	0.221	0.06 (− 0.05, 0.16)	0.306
	IL-6	0.07 (− 0.05, 0.19)	0.238	0.09 (− 0.03, 0.21)	0.122
	Cortisol	0.08 (− 0.03, 0.19)	0.138	0.08 (− 0.03, 0.19)	0.132
	DHEAS	0.03 (− 0.08, 0.14)	0.550	0.05 (− 0.06, 0.16)	0.386
	TNF	0.05 (− 0.06, 0.16)	0.406	0.05 (− 0.06, 0.16)	0.374
	IL-8	0.01 (− 0.11, 0.13)	0.891	0.03 (− 0.09, 0.15)	0.647
Walking speed	CRP	− **0.18 (**− **0.29,** − **0.07)**	**0.001**	− 0.10 (− 0.22, 0.01)	0.085
	Ad:lep ratio	**0.15 (0.04, 0.26)**	**0.008**	0.08 (− 0.04, 0.20)	0.200
	IL-10	− 0.05 (− 0.16, 0.06)	0.348	− 0.07 (− 0.18, 0.03)	0.176
	IL-6	− 0.01 (− 0.13, 0.11)	0.908	− 0.00 (− 0.12, 0.12)	0.965
	Cortisol	− 0.02 (− 0.14, 0.09)	0.720	− 0.06 (− 0.17, 0.06)	0.334
	DHEAS	− 0.01 (− 0.12, 0.10)	0.848	− 0.01 (− 0.12, 0.10)	0.875
	TNF	0.01 (− 0.10, 0.12)	0.898	− 0.01 (− 0.12, 0.10)	0.894
	IL-8	− 0.04 (− 0.16, 0.08)	0.478	− 0.04 (− 0.16, 0.08)	0.541
Appendicular lean mass	CRP	**0.13 (0.02, 0.25)**	**0.025**	**0.15 (0.05, 0.25)**	**0.002**
	Ad:lep ratio	− 0.11 (− 0.23, 0.00)	0.055	− 0.05 (− 0.15, 0.05)	0.306
	IL-10	0.04 (− 0.07, 0.16)	0.480	0.03 (− 0.06, 0.13)	0.503
	IL-6	− 0.00 (− 0.13, 0.13)	0.984	− 0.07 (− 0.18, 0.04)	0.201
	Cortisol	− **0.12 (**− **0.24,** − **0.00)**	**0.042**	− **0.10 (**− **0.20,** − **0.01)**	**0.039**
	DHEAS	− **0.13 (**− **0.25,** − **0.01)**	**0.032**	− 0.08 (− 0.18, 0.02)	0.107
	TNF	− 0.02 (− 0.14, 0.09)	0.700	− 0.06 (− 0.15, 0.04)	0.227
	IL-8	− **0.19 (**− **0.32,** − **0.06)**	**0.005**	− **0.11 (**− **0.22,** − **0.00)**	**0.043**
EWGSOP sarcopenia^b^	CRP	1.37 (0.90, 2.08)	0.144	**1.77 (1.08, 2.90)**	**0.023**
	Ad:lep ratio	0.86 (0.61, 1.21)	0.387	**0.62 (0.44, 0.87)**	**0.006**
	IL-10	1.06 (0.74, 1.51)	0.761	0.99 (0.74, 1.34)	0.969
	IL-6	1.44 (0.78, 2.68)	0.246	1.27 (0.71, 2.28)	0.416
	Cortisol	**1.39 (1.08, 1.79)**	**0.011**	1.35 (0.95, 1.91)	0.092
	DHEAS	1.12 (0.75, 1.68)	0.576	1.11 (0.74, 1.64)	0.619
	TNF	1.32 (0.87, 2.02)	0.194	1.31 (0.89, 1.94)	0.167
	IL-8	**1.57 (1.35, 1.84)**	**<** **0.001**	**1.37 (1.10, 1.71)**	**0.005**

Separate regression models were fitted for each baseline inflammatory predictor

Sex-specific SD scores were derived for inflammatory predictors and continuous outcomes. Apart from IL-8, all inflammatory markers were log-transformed prior to standardising

Unadjusted estimates correspond to Pearson correlations between inflammatory markers and outcomes (apart from sarcopenia)

Change in grip strength from HCS baseline to follow-up was obtained using a residual change method to ensure grip change measure was independent of baseline grip level

*Ad:lep ratio* adiponectin:leptin ratio, *EWGSOP* European Working Group on Sarcopenia in Older People

Significant associations (*p* **<** 0.05) are in bold

^a^Regression models adjusted for the following characteristics at baseline: gender, age, follow-up time, height, weight-for-height residual, smoking history (ever vs never), alcohol consumption, diet quality and physical activity. Models for appendicular lean mass were not adjusted for weight-for-height residual and models for change in grip were not adjusted for follow-up time (follow-up time was already used to derive the grip change measure)

^b^Estimates are relative risks per SD increase in baseline inflammatory predictors. Relative risks were derived using Poisson regression models with robust variance estimation

### Associations Between Baseline Inflammation Markers and Sarcopenia at Follow-Up

Lower adiponectin:leptin ratios and elevated levels of CRP, cortisol and IL-8 were associated with increased risk of sarcopenia as defined by the EWGSOP algorithm at follow-up, although the strength of associations varied according to the other covariates included in the models.

## Discussion

Using data from the HCS, we have shown that indices of inflammation are associated with each of the three components of sarcopenia; muscle mass, muscle strength and physical performance. The strongest associations were with CRP and were robust to adjustment for gender and lifestyle characteristics. Additional relationships were found with IL-8, a principal member of the inflammatory cascade, and cortisol, the most potent of the endogenous immunosuppressants. Fully-adjusted associations were similar when additionally adjusted for number of systems medicated (a marker of comorbidity) or steroid use. This study makes an important contribution to the published literature by examining the longitudinal associations between a wide range of inflammatory markers, cytokines and adipokines, with measures of muscle mass, strength, physical performance, and sarcopenia.

Our study has some limitations. First, selection bias may have affected our study at several stages. In common with many epidemiological studies, a healthy responder bias has been observed in HCS [11], however, baseline participants remained broadly comparable with participants in the nationally representative Health Survey for England [11]. In addition, sample attrition across the various waves of follow-up (possibly owing to mortality, morbidity and changes of address) could have resulted in further selection effects. However, we have described participant characteristics according to inclusion status across the study and found no major differences. Furthermore, because our analyses were internal, unless there was a systematic difference in the associations of interest among our study participants and those who were invited to take part in the study but did not, no major bias should have occurred. Secondly, although a relatively small sample size was used, the similarity between our findings and those of similar studies is encouraging [8, 16]. Thirdly, the markers of inflammation were measured at a single time point, which could be influenced by concurrent illnesses. However, due to the arrangement of the research clinics, it is unlikely that participants would have attended if acutely unwell or debilitated by long-term chronic disease. This is supported by that fact that the majority of the inflammation markers were within the normal range, suggesting that this was a largely healthy population. It can, therefore, be assumed that the inflammatory markers represent the inflammatory phenotype of the participant within the cohort as a whole. Fourthly, it is possible that the serum measurements of cytokine levels may not be indicative of the cytokine milieu at the level of the tissue of interest; muscle. Further in vivo, tissue-level studies are required to investigate this further. Finally, the availability of outcomes (other than grip strength) at baseline would have enabled investigation of the association between inflammation and change in muscle mass and function.

Our study also has many strengths. First, a residual change method was used to calculate a measure of change in grip strength which was independent of baseline grip strength and free from the effect of regression to the mean. Second, we examined the longitudinal associations between a broad panel of inflammatory markers and several defining components of sarcopenia with adjustment for a wide range of potential confounders (however, we cannot rule out the potential effects of unmeasured confounders). Finally, the HCS provides a strong foundation for research owing to: conduct of the fieldwork according to strict protocol by trained research nurses; detailed phenotyping of the cohort; and the continued management of the study by an experienced multidisciplinary team.

C-reactive protein is a consummate marker of inflammation, utilised ubiquitously in clinical practice and has been much studied in relation to many conditions. It is produced by the liver and rises up to 10,000-fold in response to major tissue damage or severe infection [17]. However, outside of acute stimuli, the year to year, intra-individual variation in CRP is stable [18] with a significant association between baseline CRP and risk of vascular disease and mortality from cardiovascular disease, cancer and respiratory conditions [19]. This supports our use of a single measurement of inflammatory marker as a surrogate for overall, inflammatory phenotype. We found that a higher CRP was associated with lower grip strength, accelerated loss of grip strength, slower gait speed (in gender-adjusted analyses only), and sarcopenia (in fully-adjusted analyses only). Similar associations between reduced physical performance and grip strength and increasing CRP have been demonstrated in elderly Italian [8] and Dutch [20] cohorts. Further replication is provided by a previous study which demonstrated a graded association between increased CRP and reduced grip strength [21]. A similar, graded relationship between reduced CRP and faster gait speed has been seen in a multi-national study involving 64-74 year old participants from the North and South American continents [16]. This would support our finding of a negative association between CRP and walking speed.

The mechanism by which the association of CRP with parameters of muscle strength and function occurs is still under investigation though, a primed, pro-inflammatory environment, which would result in an increase in CRP as a marker of inflammation, is associated with the process of ageing, frailty and sarcopenia [22].

A raised CRP was also associated with greater ALM. This may seem counterintuitive, as a higher inflammatory burden would inhibit the anabolic processes involved in muscle tissue formation. However, if we consider that those with greater fat mass are also likely to have greater muscle mass (as demonstrated by ALM), a raised CRP would represent the increased inflammatory burden associated with adipose tissue. This is supported by the fact that CRP was not associated with ALM index when adjusted for whole body fat mass index in our sample (data not shown). Indeed, as adiposity increases, adipose tissue dysfunction can occur. Lipids leak into the circulation and deposit in muscle tissue leading to the localised production of reactive oxygen species and an inflammatory response (the latter of which will lead to a raised CRP). This can result in damage to mitochondria, muscle tissue damage, and thus, reduced muscle function.

Adipose tissue, besides its primary function as energy storage, also plays significant roles in the determination of the prevailing balance between pro- and anti-inflammatory states in the body as a whole. Adiponectin, an anti-inflammatory adipokine, inhibits nuclear factor κB (NFκB) leading to reduced macrophage activation, reduced TNF and interferon gamma (IFNγ) and increases IL-10 and interleukin-1 receptor antagonist (IL-1RA). It is negatively associated with fat mass and leptin [23], and levels reduce with age [24]. On the other hand, leptin, a pro-inflammatory adipokine, reflects adipose mass [25] and is associated with the pro-inflammatory mediators; TNF, IL-6, interleukin-12 (IL-12) and activates natural killer lymphocytes [26]. In our study, we found that an increased adiponectin:leptin ratio was associated with faster walking speed (though this association was not robust beyond gender-adjustment) and with reduced risk of sarcopenia. This is explained by the fact that a higher adiponectin:leptin ratio purports to represent a more anti-inflammatory adipokine profile, which would fit with a reduced propensity to muscle ageing and damage.

The negative associations of cortisol and IL-8 with ALM are simply explained.

IL-8 is a rife chemotactic agent for neutrophils and higher levels will represent a more primed, and active, innate immune response. Neutrophil dysregulation, including a reduced susceptibility to apoptotic signals and a more limited response to chemotactic agents, occurs with increasing age [27]. This is important in the process of inflammaging (a low-level state of inflammation as a result of an aged immune system) as delayed neutrophil transit and increased dysregulation leads to the leakage of matrix metalloproteinases and other enzymes leading to localised tissue damage. If this process was to occur within muscle, we would see a raised IL-8 in association with sarcopenia. Certainly, an active immune response has previously been associated with cachexia and frailty [22], so an association with sarcopenia would be aetiologically coherent.

In order to counteract inflammaging, the body emboldens the process of ‘anti-inflammaging’ in the form of anti-inflammatory cytokine production and cortisol release. Cortisol is the most potent, endogenous immunosuppressive agent and production can be stimulated by interleukin-1 (IL-1), IL-6 and TNF via the hypothalamo-pituitary axis. However, as well as the anti-inflammatory effects [28], it has direct effects of bone resorption, gluconeogenesis and muscle catabolism. Higher levels of cortisol (as seen in the cortisol to DHEAS ratio) have been associated with significantly increased odds of frailty at 10-year follow-up [29]. Thus, the association we observed between higher cortisol and reduced ALM makes physiological sense and could be demonstrative of an increased anti-inflammatory, ‘anti-inflammaging’ response.

In summary, our findings highlight an important role for CRP, IL-8 and other elements of immune function in the development of sarcopenia and its constituent components; muscle strength, muscle mass and physical function. Further work is required to delineate these relationships.
